# Formulation and Characterization of Doxycycline-Loaded Polymeric Nanoparticles for Testing Antitumor/Antiangiogenic Action in Experimental Colon Cancer in Mice

**DOI:** 10.3390/nano12050857

**Published:** 2022-03-03

**Authors:** Reem Alshaman, Abdullah Alattar, Rehab M. El-Sayed, Ahmed R. Gardouh, Rabie E. Elshaer, Amany Y. Elkazaz, Mohamed Ahmed Eladl, Mohamed El-Sherbiny, Noha E. Farag, Ahmed Mohsen Hamdan, Sawsan A. Zaitone

**Affiliations:** 1 Department of Pharmacology and Toxicology, Faculty of Pharmacy, University of Tabuk, Tabuk 71491, Saudi Arabia; ralshaman@ut.edu.sa (R.A.); aalattar@ut.edu.sa (A.A.); 2 Department of Pharmacology and Toxicology, Faculty of Pharmacy, Sinai University, El-Arish 45511, Egypt; rehab.mahmoud@su.edu.eg; 3 Department of Pharmaceutics and Industrial Pharmacy, Faculty of Pharmacy, Suez Canal University, Ismailia 41522, Egypt; ahmed_mahmoud@pharm.suez.edu.eg; 4 Department of Pharmacy, Faculty of Pharmacy, Jadara University, Irbid 21110, Jordan; 5 Department of Pathology, Faculty of Medicine, Al-Azhar University, Cairo 11651, Egypt; ra88871@azhar.edu.eg; 6 Biochemistry and Molecular Biology Department, Faculty of Medicine, Suez Canal University, Ismailia 41522, Egypt; amany_elkazaz@med.suez.edu.eg; 7 Biochemistry and Molecular Biology Department, Faculty of Medicine, Port Said University, Port Said 42526, Egypt; 8 Basic Medical Science Department, University of Sharjah, Sharjah 27272, United Arab Emirates; 9 Department of Basic Medical Sciences, College of Medicine, AlMaarefa University, Riyadh 71666, Saudi Arabia; msharbini@mcst.edu.sa; 10 Department of Physiology, Faculty of Medicine, Suez Canal University, Ismailia 41522, Egypt; nohafarag@gmail.com; 11 Department of Physiology, College of Medicine, Taif University, Taif 21974, Saudi Arabia; 12 Department of Pharmacy Practice, Faculty of Pharmacy, University of Tabuk, Tabuk 71491, Saudi Arabia; a_hamdan@ut.edu.sa; 13 Department of Pharmacology and Toxicology, Faculty of Pharmacy, Suez Canal University, Ismailia 41522, Egypt

**Keywords:** angiogenesis, mouse colon cancer, eudragit S100, hydroxypropyl methylcellulose phthalate, doxycycline polymeric nanoparticles, nanoprecipitation method

## Abstract

Nanotherapeutics can enhance the characteristics of drugs, such as rapid systemic clearance and systemic toxicities. Polymeric nanoparticles (PRNPs) depend on dispersion of a drug in an amorphous state in a polymer matrix. PRNPs are capable of delivering drugs and improving their safety. The primary goal of this study is to formulate doxycycline-loaded PRNPs by applying the nanoprecipitation method. Eudragit S100 (ES100) (for DOX-PRNP1) and hydroxypropyl methyl cellulose phthalate HP55 (for DOX-PRNP2) were tested as the drug carrying polymers and the DOX-PRNP2 showed better characteristics and drug release % and was hence selected to be tested in the biological study. Six different experimental groups were formed from sixty male albino mice. 1,2,-Dimethylhydrazine was used for 16 weeks to induce experimental colon cancer. We compared the oral administration of DOX-PRNP2 in doses of 5 and 10 mg/kg with the free drug. Results indicated that DOX-PRNP2 had greater antitumor activity, as evidenced by an improved histopathological picture for colon specimens as well as a decrease in the tumor scores. In addition, when compared to free DOX, the DOX-PRNP2 reduced the angiogenic indicators VEGD and CD31 to a greater extent. Collectively, the findings demonstrated that formulating DOX in PRNPs was useful in enhancing antitumor activity and can be used in other models of cancers to verify their efficacy and compatibility with our study.

## 1. Introduction

Nanotherapeutics is a developing field within the field of nanomedicine and it aims to enhance the characteristics of drugs [1]. There are various types of nanotherapeutics such as solid-lipid nanoparticles, gold nanoparticles and polymeric nanoparticles (PRNPs) [2,3,4,5].

PRNPs improve solubility, bioavailability and retention time of drugs and enhance their therapeutic effect [6,7,8,9]. PRNPs have advantages over conventional drugs in that they increase drug efficacy, protect the drug from degradation when in contact with biological fluids and control the drug release [10] due to their advantages resulting from their tiny size. PRNPs have gained a lot of attention in recent years [11,12]. PRNPs are composed of amorphous drugs dispersed within a polymer matrix [13]. Controlling drug release in the gastrointestinal tract is possible with pH-sensitive materials that release the drug selectively in the small intestine adjacent to the absorption location [14,15]. PRNPs can be utilized to be loaded with various types of medications for treatment of different types of cancer [16,17].

Cancer is an important cause of death in the world [18]. Obstacles for cancer therapy are the toxicity of drugs, multidrug resistance, and low drug uptake by cancer cells [19]. Colorectal cancer is in second place for women-diagnosed cancers and in third place for men-diagnosed cancers [20,21]. Angiogenesis is the generation of capillary blood vessels [22] that is stimulated in tumor growth and metastasis [23]. Capillaries are required to supply nutrients and oxygen to a growing tumor. Vascular endothelial growth factor (VEGF) is a key factor in angiogenesis that enhances microvascular hyperpermeability [24].

In cancer therapy, the critical question is how to achieve the desired level of chemotherapy in tumors [25]. Contemporary cancer treatment modalities continue to face significant challenges, including low drug concentrations at the tumor areas, intolerable adverse effects and development of chemo-resistance [26]. These and other constraints have mandated development of the recent advancements in cancer therapeutics.

Doxycycline (DOX) is a wide-spectrum antibiotic that is a tetracycline derivative [27] that was first approved by the FDA in the 1960s [28,29]. It is mainly used in the treatment of acne and acne rosacea [30]. This side effect of DOX was repurposed as a therapeutic effect in treatment of cancer by inhibition of mitochondrial biogenesis in cancer cells [31,32]. Moreover, it may potentially be harmful to healthy cells due to enzymatic inhibition or changes in protein synthesis [33]. The use of nanotechnology is one possible solution to this challenge [34] and to improving the efficiency of chemotherapeutic agents [35].

Since the stability of drugs can be improved utilizing pH-sensitive nanoparticles. The primary goal of this study is to formulate pH-sensitive PRNPs loaded with DOX using the nanoprecipitation technique and the polymers eudragit S100 (ES100) or hydroxypropyl methyl cellulose phthalate HP55 (HPMCP HP55) and to test their therapeutic potential against experimentally induced colon cancer in mice.

## 2. Materials & Methods

### 2.1. Synthesis of Doxycycline Polymeric Nanoparticles

#### 2.1.1. Materials

Poly(methacrylic acid, methyl methacrylate) 1:2 (Eudragit S100^®^) was kindly provided by Heinrich’s Commercial Agency (EVONIK, batch # B101205222, Cairo, Egypt) (ES100). Cellulose, 2-hydroxypropyl methyl ether and phthalic acid ester were purchased from Shin-Etsu Chemical Co. (Tokyo, Japan). HPMCP HP55 was supplied by Acino Pharm (batch #0000168267, Acino International AG Thurgauerstrasse 36/38 CH-8050, Zurich, Switzerland). α-Hydro-ω-hydroxypoly (oxyethylene) poly (oxypropylene) poly (oxyethylene) block copolymers (Poloxamer 407^®^) were obtained from BASF, (Cairo, Egypt, batch #WPHF555C). Acetone was purchased from El-Nasr chemical company (Qalyub, Egypt, batch #2013/7). Doxycycline helicate was a gift from Tabuk Pharmaceutical Company (Tabuk, KSA).

#### 2.1.2. Formulation of DOX-PRNPs with Full Characterization

**Figure 1:**
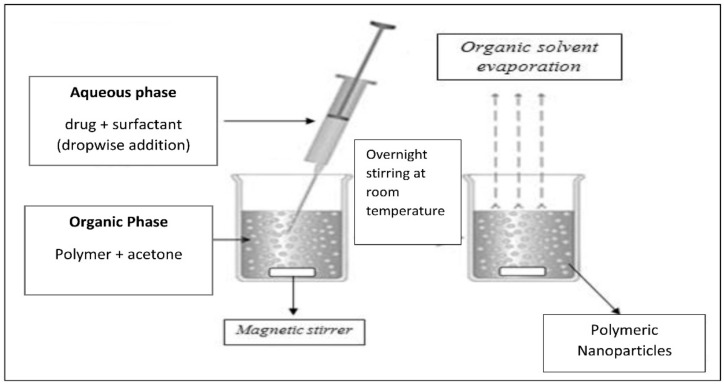
Formulation of doxycycline polymeric nanoparticles.

Preparation of polymeric nanoparticles containing doxycycline with full characterization was performed according to [36], who prepared DOX-PRNPs with hydroxypropyl methyl cellulose polymer and Tween 80 as surfactant utilizing different ratios, with little modification. Briefly, PRNPs formulations were prepared by the nanoprecipitation technique [37] for hydrophilic and hydrophobic drug encapsulation in polymer nanoparticles. We used 0.8 g % of the polymer, eudragit S100^®^ (PRNP1) or HPMCP HP55^®^ (PRNP2), and 1% Poloxamer 407^®^ as surfactant. Drop wise addition of aqueous phase with drug and surfactant added to a certain concentration of polymer dissolved in acetone (water-miscible organic solvent) was used for forming an organic phase (the final organic: aqueous phase ratio equals 1:8) as shown in Figure 1. The mixture was stirred at room temperature until turning into a milk-like mixture [38] and was left overnight with continuous stirring [39]. The drug polymer ratio in final formulations was 2:1. The efficiency of encapsulation and loading capacity were tested by an indirect method. The amount of the non-capsulated drug in the supernatant was estimated spectrophotometrically at 269 nm, utilizing an ultraviolet spectrophotometer (Shimadzu, Yokohama, Japan). Different characterizations such as zeta potential (ZP), particle size as well as the polydispersity index (PDI) of the formulated nanoparticles were measured using a Zetasizer (Malvern Instruments Ltd., Malvern, UK) [40,41], while morphology of the surface of the nanoparticles was confirmed using transmission electron microscopy (TEM) (model JTEM-2100, Tokyo, Japan) [42]. Release of the drug from different trials was carried out using dialysis bag method [43] at simulated gastric fluid containing 0.1 N HCl and final pH equalsing 1.2 for 2 h and then substituted with phosphate buffer (pH = 6.8) for the next 10 h. Temperature was kept at 37 ± 1 °C, and drug was assayed spectrophotometrically at 269 nm (UV-visible spectrophotometer, Shimadzu, Yokohama, Japan) [44]. Detailed ratios of drug to polymer with surfactant concentration and full characterizations are listed in Table 1. From these data, PRNP2 showed better characteristics and was selected to be tested for biological activity in the in vivo study.

### 2.2. In Vivo Antitumor Activity

#### 2.2.1. Animal Environment

Swiss albino mice with body weights ranging from 20–30 g were utilized. Mice were placed in plastic cages in groups of six with normal light–dark cycle and free access to drinking water and food. The animal study was approved by the institutional research ethics committee (#201907RA3).

#### 2.2.2. Experimental Design

1,2, dimethyl hydrazine (Di-MH, Sigma-Aldrich, St. Louis, MO, USA) was employed to induce experimental colon cancer in mice and was administered (10 mg/kg/week, subcutaneously) for sixteen weeks following a previously reported schedule [45,46]. Groups of mice were randomized as follows:

Group i: mice were treated with subcutaneous injections of the vehicle (saline).

Group ii: mice were subjected to induction of colon cancer by a weekly dose of Di-MH (10 mg/kg, s.c.).

Group iii, iv (free DOX 5 and 10 mg/kg): mice were subjected to Di-MH injections in the same above-mentioned schedule and treated daily with DOX (5 and 10 mg/kg, s.c.), starting from week 13 until the end of week 17.

Group iv, v (DOX-PRNP2 5 and 10 mg/kg): mice were subjected to Di-MH injections in the same above-mentioned schedule and treated daily with DOX (5 and 10 mg/kg, s.c.) starting from week 13 until the end of week 17. In general, drugs were given daily at 8:00 h. Control group received the vehicle of DOX daily parallel to doses in group iii–vi.

#### 2.2.3. Sacrification and Colon Dissection

Mice were subjected to thiopental anesthesia and killed by cervical dislocation at the end of the experiment. Tissue samples were taken from the descending colon and preserved in 10% phosphate-buffered formalin (pH 7.4, molecular formula CH_2_O) overnight and then embedded in paraffin wax [47].

#### 2.2.4. Histopathological Staining and Examination

Following fixation in formalin, colon specimens were fixed in paraffin, and 4 μM slices were cut for further processing using blocks. Specimens were stained with hematoxylin and eosin (H&E) and examined microscopically in a blinded manner for differential structural alterations. Each group was evaluated on three distinct sections. Intermediate magnification (100×) and high magnification (400×) images were captured using an Olympus^®^ digital camera.

Histologic scoring was used to determine the degree of dysplasia using previously stabilized parameters. In summary, tissue specimens were classified as having no dysplastic changes or having dysplastic changes: non-dysplasia—goblet cells that appear normal (basal-oriented nuclei and apical localization of mucus); mild dysplasia—hypercellularity of elongated cells and localized nuclear stratification were observed (hyperchromasia) [48].

Moderate dysplasia is characterized by hypercellularity in elongated cells with basophilic cytoplasm, expanded vesicular nuclei, conspicuous nucleoli, and loss of mucosal architecture in the crypts as well as severe focal lesions with nuclear stratification, anomalies in the structure of the crypts, and a loss or decrease in the number of goblet cells [49]. Additionally, the percentage of crypts with normal architecture or with mild, moderate, or severe dysplastic alterations was assessed and averaged for each experimental group.

#### 2.2.5. Immunohistochemical Staining for VEGF

After antigen retrieval, 5% normal goat serum was applied to the slides for 45 min for preventing non-specific antibody binding. At 4 °C overnight, slides were treated with the desired primary antibodies. At a dilution of 1:100, primary rabbit polyclonal antibodies against VGEF (diluted 1:100, #A17877, ABclonal, Woburn, MA, USA) was employed. Slides were cleaned, treated with secondary antibody, washed again, and then covered with DAB and counterstained. Finally, slides were blindly viewed under an Olympus microscope. The images were captured at magnifications of 100× and 400× using an Olympus^®^ built-in digital camera. The percentage of stained area was determined using ImageJ (NIH, Bethesda, MD, USA), and the percentages were digitalized to obtain the area of immunostaining.

#### 2.2.6. Western Blot Analysis

In RIPA buffer containing protease and phosphatase inhibitors, isolated colon tissues were homogenized. Homogenates were centrifuged at 14,000× *g* for 20 min at 4 °C to remove insoluble material. Transferring the supernatant to a new microcentrifuge tube, 5 μL was used to determine the protein concentration using the Bio-Rad Quick Start^TM^ Bradford Protein Assay kit. After initial denaturation with 4x Laemmli Sample Buffer, similar amounts of protein from colon tissues homogenate were loaded onto sodium dodecyl sulfate polyacrylamide gel (Bio-Rad, Hercules, CA, USA). Following electrophoresis separation of proteins, the proteins from the gels were transferred to nitrocellulose membranes. Incubation in 5% nonfat dried milk blocking the free sites on the membranes (Bio-Rad, Hercules, CA, USA) were performed for 1 h. This was followed by washing of the blocked membranes and incubating them with primary antibodies specific for the targeted proteins: a rabbit recombinant polyclonal anti-cluster of differentiation 31 (CD31) antibody (RM1006) (ab281583 Abcam) at 1/1000 dilution, a mouse monoclonal antibody to VEGF (sc-7269) from Santa Cruz Biotechnology Inc. (Santa Cruz, CA, USA) at dilution 1:200 and antibody to β-actin (sc-8432) (SantaCruz Biotechnology, Santa Cruz, CA, USA) overnight at 4 °C with gentle agitation. Following that, the blots were washed and incubated with the appropriate secondary antibody conjugated to horseradish peroxidase (HRP) and goat anti-mouse, followed by enhanced chemiluminescence detection using the enhanced chemiluminescence ECL Advance^TM^ Western blotting detection kit. Densitometry was used to quantify the intensity of immunoreactivity using the ImageJ software (NIH, Bethesda, MD, USA).

#### 2.2.7. Enzyme Linked-Immunosorbent Assay for the Proangiogenic Factors

The colon homogenates were assayed for IL-6, TNF-α and VEGF using ELISA kits: mouse IL-6 ELISA kit (Sunred Biological Technology Company, Shanghai, China), mouse TNF-α ELISA kit (Cloud-Clon Crop Company, Katy, TX, USA) and mouse VEGF ELISA kit (Cloud-Clon Crop Company, Katy, TX, USA). The optical density of the reactions was measured at 450 nm.

### 2.3. Statistical Analysis

The statistical tests were conducted using the social sciences statistical package. (SPSS Software, SPSS Inc., Chicago, IL, USA). Finally, the differences were considered significant in any of the statistical tests when *p* < 0.05. The Shapiro–Wilks test was used to determine the normality of the data distribution. For data with a Gaussian distribution, one-way analysis of variance, ANOVA, was applied for analyzing the measurements. Additionally, post hoc analysis was used to compare the study groups. The Mann-Whitney U test was used to examine data with a non-Gaussian distribution. Two-tailed data are expected.

## 3. Results

### 3.1. Polymeric Nanoparticles Characterizations

**Figure 2:**
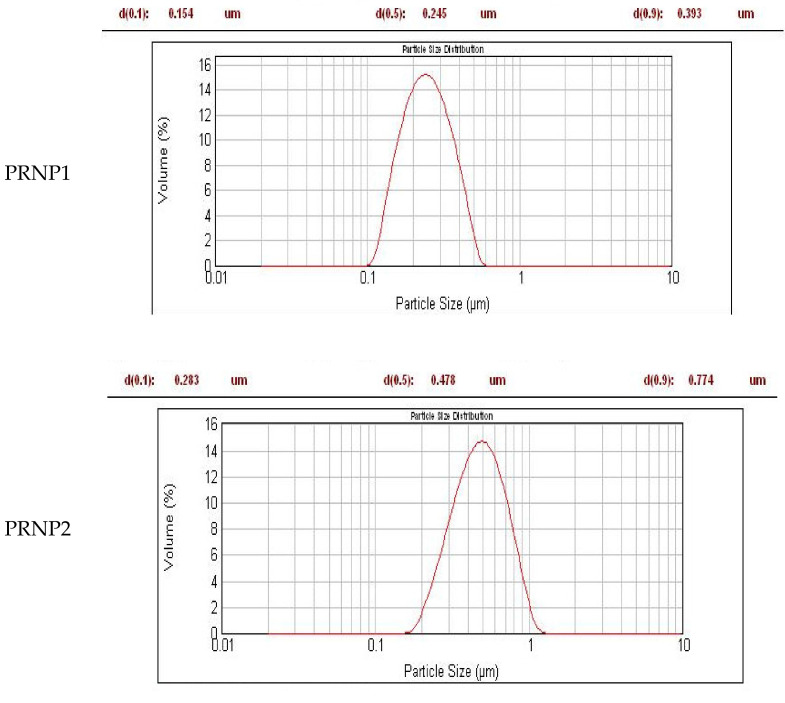
Particle size by laser diffraction technique of prepared polymer nanoparticles of doxycycline prepared by nanoprecipitation technique (displaced for better comparison).

**Figure 3:**
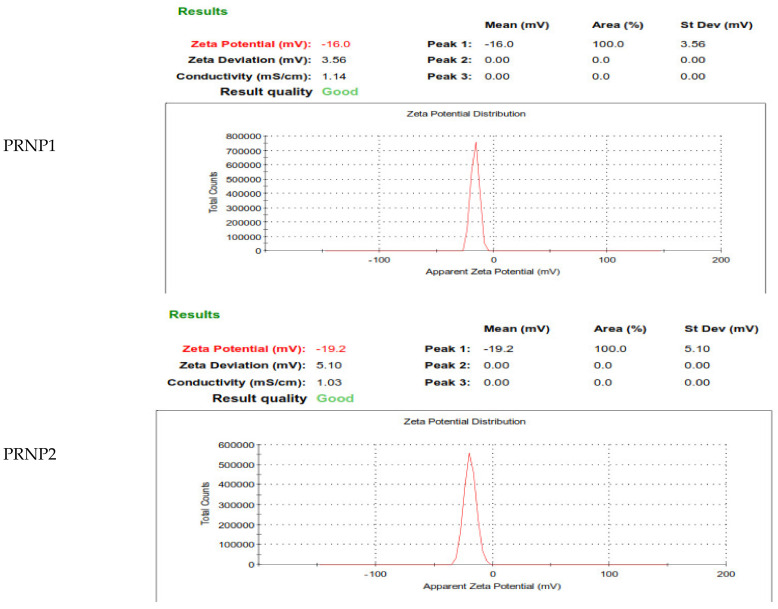
Zeta potential values for the prepared polymer nanoparticles of doxycycline prepared by nanoprecipitation technique.

**Figure 4:**
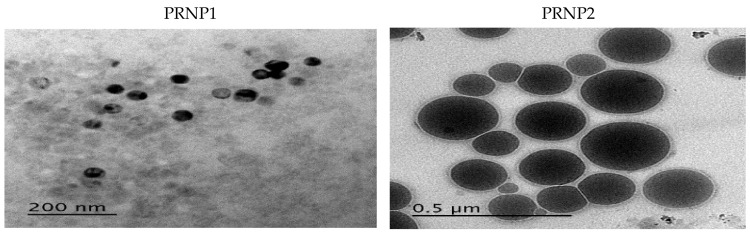
Transmission electron micrographs of prepared polymer nanoparticles of doxycycline prepared by nanoprecipitation technique, showing the particle size of prepared nanoparticles (displaced for better visualization).

Nanoprecipitation technique was suitably used for preparation of pH responsive DOX-PRNPs to control its release and modulate its pharmacodynamic influence. Figure 2 and Figure 3 show that particle size of formulated nanoparticles were below micrometers and showed moderate stability owing to values of ZP (below 30 mV). Loading capacity of PRNP2 was higher than that of PRNP1 while maintaining the same formulation conditions. PDI, which is an indicator of uniformity of prepared particle size, showed a value below 0.5, indicating homogeneity and monodisperse feature. The TEM micrographs of the prepared nanoparticles seemed spherical with a smooth surface as shown by Figure 4.

**Figure 5:**
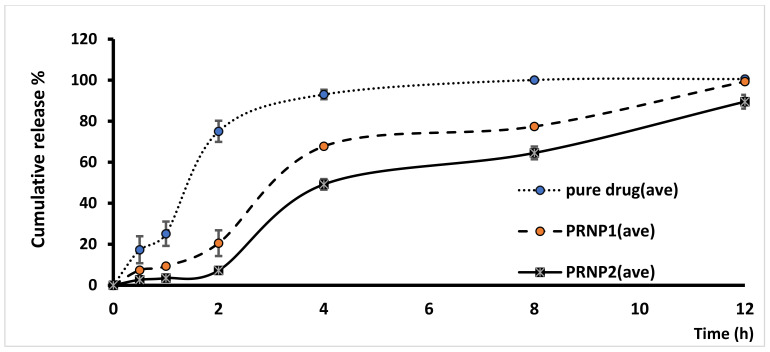
Release profile of doxycycline from prepared polymeric nanoparticles prepared by nanoprecipitation technique.

The prepared PRNPs revealed negative ZPs, which gives an indication about stability due to low tendency for aggregation. Upon studying DOX release form prepared PRNPs, results showed that the type of the polymer significantly impacts drug release, where PRNP1 presented greater release than PRNP2 (Figure 5). Further, PRNP2 dissolved at greater pH than that of PRNP1, and the two formulations showed more delayed release than the pure drug.

### 3.2. Biological Activity against Colon Cancer

#### 3.2.1. Histopathological Examination

**Figure 6:**
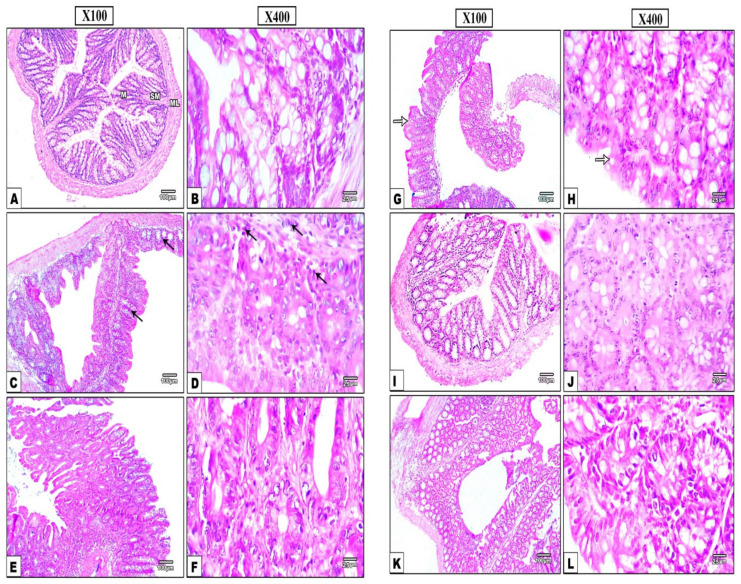
Hematoxylin and eosin stained colonic sections representing the histopathological changes. (**A**,**B**) show normal mucosal layers (M), submucosa (SM) and musculosa (ML) of saline group. Mucosal surface shows integral columnar epithelium, acidophilic cytoplasm and basal oval nuclei, flask-shaped goblet cells with vacuolated cytoplasm and flattened nuclei at the base (White arrow), crypts with normal shape and narrow openings, and between crypt normal connective tissue of lamina propria. (**C**,**D**) show hyperplastic distorted mucosa of Di-MH group lined by improperly packed cells having hyperchromatic nuclei (black arrow), disintegrated goblet cell, morphologically altered crypts, and lamina propria with inflammatory cell infiltration. (**E**,**F**) show distorted architecture in DOX 5 mg/kg treated group with partial moderate (grade 2) dysplasia, numerous goblet cells, and mild inflammatory cell infiltration in between crypts. DOX 10 mg/kg group (**G**,**H**) display partial moderate (grade 2) dysplasia, and moderate number of goblet cells. DOX-PRNP2 5 mg/kg (**I**,**J**) and 10 mg/kg group (**K**,**L**) display a nearly normal mucosal architecture with unaltered crypts, and submucosa. Magnifications of H&E stain X100 bar 100, X400 bar 25.

As shown in Figure 6, the histopathological examinations of the colon tissues from saline group (Figure 6A,B) stained with H&E showed the normal mucosa, submucosa and musculosa. The mucosa showed columnar epithelium with intact surface, acidophilic cytoplasm, and basal oval nuclei. Crypts show normal shape with narrow openings, while goblet cells are flask in shape with basal flattened nuclei and vacuolated cytoplasm. Conversely, the Di-MH control group (Figure 6C,D) showing hyperplasia with irregular shaped mucosa and slightly raised showed lining by inappropriate crowded hyperchromatic nuclei, distortion in crypts with disintegrated goblet cell and appearance of laminar cellular infiltration. Moreover, colon tissue sections from DOX 5 mg treated group (Figure 6E,F) and DOX 10 mg group (Figure 6G,H) showed moderate dysplasia (grade 2) with higher number of goblet cells but fewer inflammatory cells. Furthermore, using the 5 mg/kg of DOX-PRNP2 (Figure 6I,J) or 10 mg/kg group (Figure 6K,L) showed an almost normal appearance for crypts, mucosa and submucosa.

**Figure 7:**
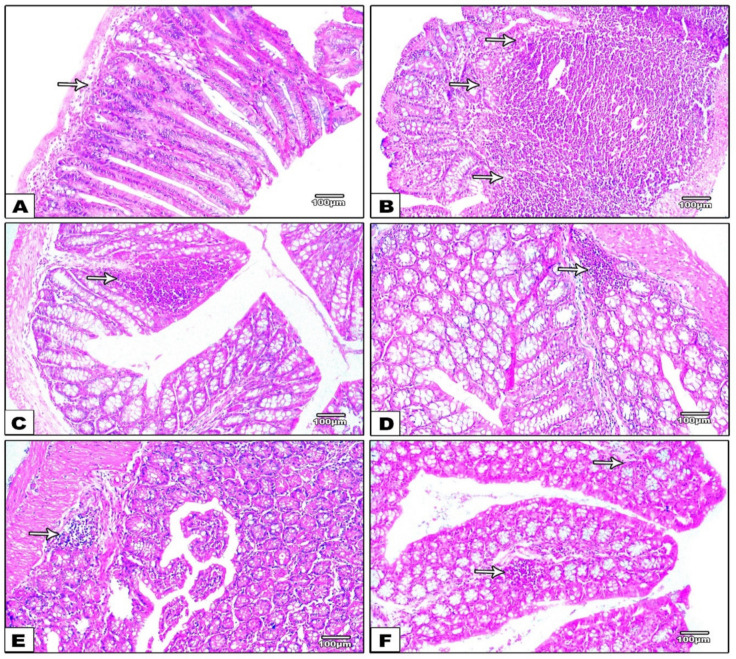
In the control negative group (**A**), microscopic images of H&E stained colonic sections show only a few scattered chronic inflammatory cells. Conversely, the Di-MH group (**B**) has a marked positive inflammatory infiltrate with prominent lymphoid follicles (arrows). Moderate inflammation with lymphoid aggregates in the DOX 5 mg/kg group (**C**), mild inflammation with minor aggregates in the DOX 10 mg/kg group (**D**), and few inflammatory cells in the DOX-PRNP2 5 and 10 mg group (**E**,**F**). Magnifications of H&E stain X100 bar 100, X400 bar 25.

Microscopic examination of stained colonic sections showed minimal scattered chronic inflammatory cells in control negative group (Figure 7A). Meanwhile, the Di-MH group (Figure 7B) showed positive dense inflammatory infiltrate with lymphoid follicles. Colonic sections from treated groups showed moderate inflammation with lymphoid aggregate in group DOX 5 mg/kg (Figure 7C), mild inflammation with mild aggregates in group DOX 10 mg/kg (Figure 7D) and few inflammatory cells in DOX-PRNP2 5 and 10 mg/kg (Figure 7E,F).

**Figure 8:**
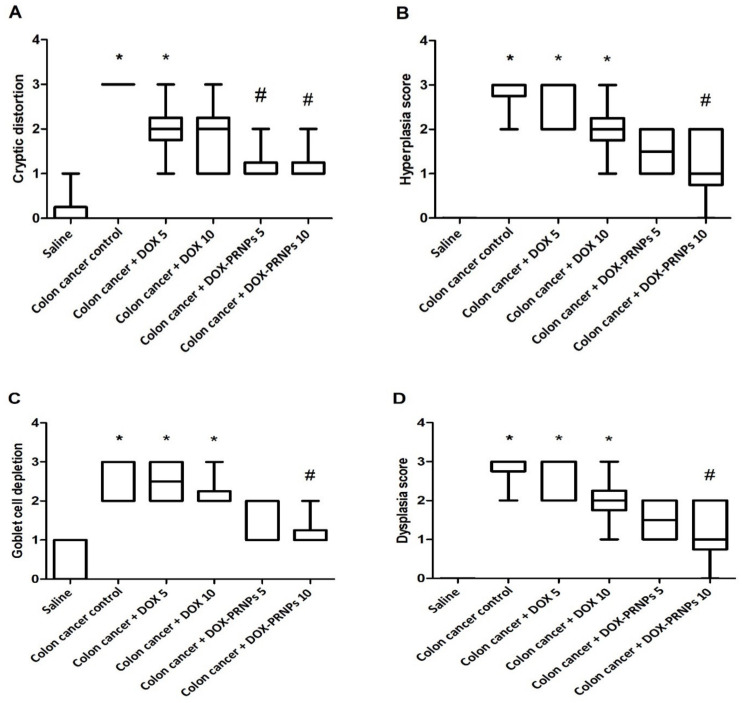
Histologic scores given to colon specimens stained with H&E. (**A**) Cryptic distortion, (**B**) hyperplasia, (**C**) goblet cell depletion and (**D**) dysplasia score. Scoring was performed for each item from 0–3 by an experienced pathologist. Data were analyzed by Kruskal-Wallis ANOVA and Dunn’s post hoc test for intergroup comparison at *p* < 0.05. *: versus saline group, #: versus colon cancer control group, *p* < 0.05. PRNPs: PRNP2 prepared from hydroxypropyl methyl cellulose phthalate HP55 as a polymer.

Figure 8 demonstrates the tumor scores given to colon specimens. Scores for cryptic distortion were greater in the colon cancer control group than in the saline group. The scores were significantly reduced in mice treated with DOX-PRNP2 5 or 10 mg/kg (Figure 8A). Scores for hyperplasia, goblet cell depletion and dysplasia were also significantly higher in the colon cancer control group compared to the saline group (Figure 8B–D); these three scores were significantly reduced in the DOX-PRNP2 10 mg/kg group.

#### 3.2.2. Immunohistochemistry for VEGF

**Figure 9:**
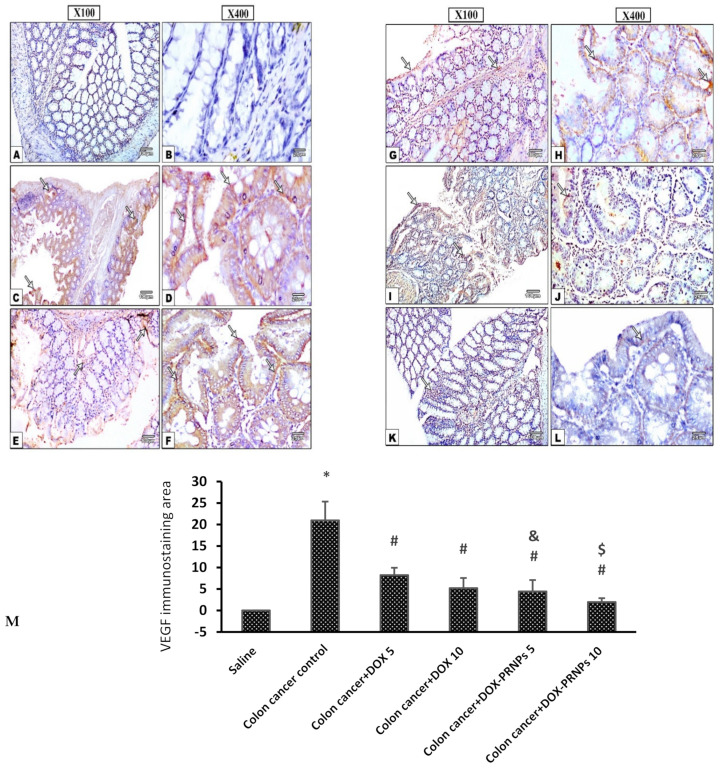
Microscopic pictures of immunostained colonic sections against VEGF. Images show negative expression of colonic mucosa in the saline group (**A**,**B**). Control group (**C**,**D**) presents marked brown positive expression in afflicted colonic mucosa of cancer colon (white arrows), while DOX 5 and 10 and DOX-PRNP2 5 and 10 mg/kg treated groups (**E**–**L**) indicate mild brown positive expression in affected mucosa (white arrows). Mayer’s hematoxylin was used as counterstain with IHC. Magnifications of VEGF immune stain X100 bar 100, X400 bar 25. (**M**) Column chart representing mean ± SDM for area % of VEGF immunostaining, *: versus saline group, #: versus colon cancer control, &: versus colon cancer + DOX 5 mg/kg and $: versus colon cancer + DOX 10 mg/kg, *p* < 0.05. PRNPs: PRNP2 prepared from hydroxypropyl methyl cellulose phthalate HP55 as a polymer.

Concerning the immunohistochemistry, the VEGF-immunostained colonic sections showed negative immune reaction in colonic mucosa of the saline group (Figure 9A,B) whereas the Di-MH-induced colon cancer control group demonstrated strong positive brown immune reaction in the affected mucosal layers (Figure 9C,D). The DOX 5 and 10 mg/kg and DOX-PRNP2 5 and 10 mg/kg treated groups showed weaker positive reactions in the affected mucosal layers (Figure 9E–L).

#### 3.2.3. Western Blot Analysis for CD31 and VEGF

**Figure 10:**
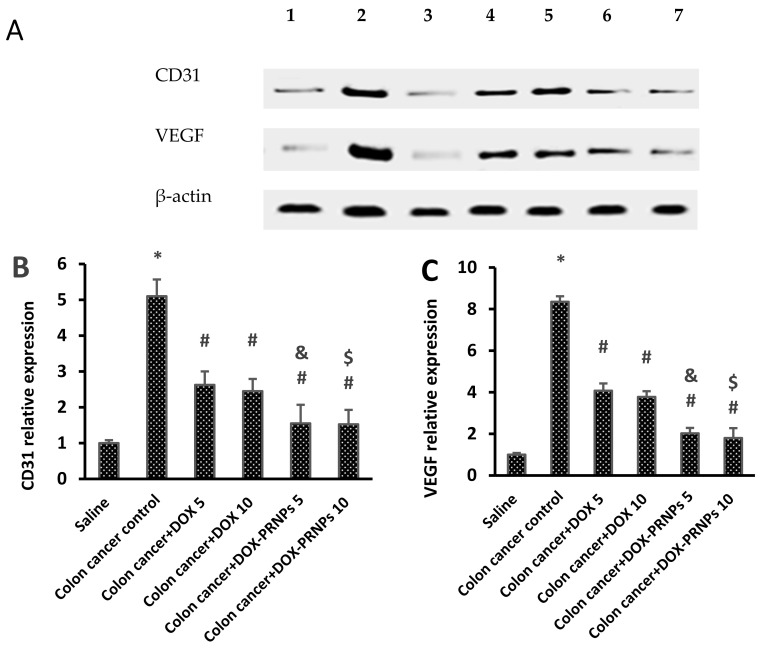
Western blotting for VEGF and CD31 in colon cancer model. (**A**) Western blots for CD31, VEGF and β-actin protein. 1: saline, 2: Di-MH, 3: saline, 4,5: Di-MH + DOX (5 and 10) mg/kg, 6,7: Di-MH + DOX-PRNP2 (5 and 10) mg/kg. (**B**,**C**) Column charts for mean ± SDM for density of CD31 and VEGF. *: versus saline group, #: versus colon cancer control, &: versus colon cancer + DOX 5 mg/kg and $: versus colon cancer + DOX 10 mg/kg, *p* < 0.05. PRNPs: PRNP2 prepared from hydroxypropyl methyl cellulose phthalate HP55 as a polymer.

Figure 10A shows the WB gels for the bands of CD31, VEGF and β-actin. Current results showed significant augmentation in CD31 and VEGF protein expression in Di-MH mice compared to control negative group (Figure 10B,C). Conversely, mice treated with DOX and DOX-PRNP2 showed a significant reduction in the protein expression compared to the Di-MH mice group. Mice treated with DOX-PRNP2 in both doses (5 and 10 mg/kg) showed further reduction versus the mice groups treated with DOX (5 and 10 mg/kg), respectively.

#### 3.2.4. Enzyme Linked Immunosorbent Assay for Proangiogenic Factors in the Colon Homogenates

**Figure 11:**
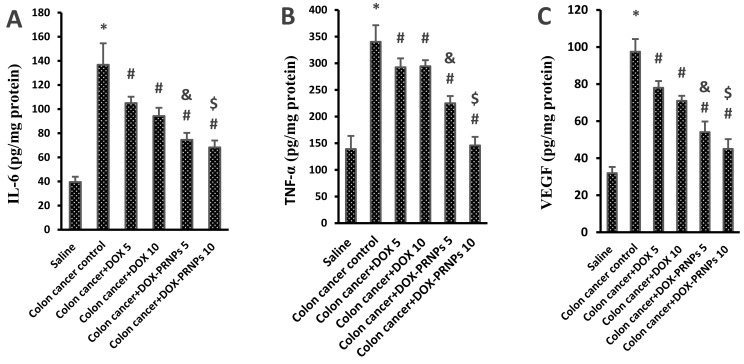
Level of the proangiogenic factors in the colon homogenates of the experimental groups. (**A**) IL-6, (**B**) TNF-α and (**C**) VEGF. *: versus saline group, #: versus colon cancer control group, &: versus colon cancer + DOX 5 mg/kg and $: versus colon cancer + DOX 10 mg/kg, *p* < 0.05. PRNPs: PRNP2 prepared from hydroxypropyl methyl cellulose phthalate HP55 as a polymer.

Figure 11 shows that the colon cancer control group demonstrated a 3.5-fold increase in IL-6 level (Figure 11A), 3.44-fold increase in TNF-α level (Figure 11B) and 3-fold increase in VEGF level (Figure 11C) compared to the saline group. Mice treated with DOX and DOX-PRNPs showed significant reductions in the level of these three proangiogenic factors compared to the colon cancer control mice. Mice treated with DOX-PRNPs in both doses (5 and 10 mg/kg) showed further reduction versus the mice groups receiving DOX (5 and 10 mg/kg) (Figure 11A–C).

## 4. Discussion

**Figure 12:**
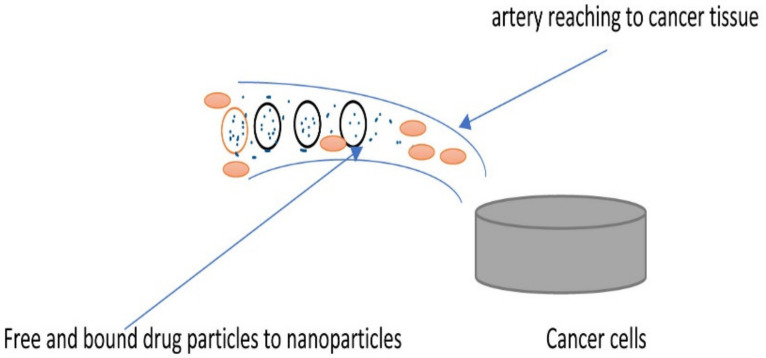
Release of drugs from nanoparticles to reach cancer cells through circulation.

Colorectal cancer is a major public serious health risk due to its prevalence and high death rates [50,51], Chemotherapies have significantly improved survival rates of individuals suffering from locally advanced stage malignancies [52,53,54]. Unfortunately, their efficacy is restricted due to cancer cells developing multidrug resistance, as well as significant side effects and dose-limiting toxicities [55,56]. Nanomedicaments are able to broadly drug load into cancer cells without depending on cell surface transporters, exterminating drug metabolism and efflux, while also reducing adverse effects associated with tissue-dependent drug uptake [16] (Figure 12).

### 4.1. Polymeric Nanoparticle Characterizations

The nanoprecipitation technique was used for preparation of pH responsive DOX-PRNPs to control its release and modulate its pharmacodynamic influence. The formulated particle size was below micrometers and showed moderate stability owing to values of ZP. The loading capacity of PRNP2 was higher than that of PRNP1; this may be due to the type of the utilized polymer. While maintaining the same formulation conditions, PRNP1 showed a smaller size than those prepared using PRNP2. This observation may take place due to the fact that “the molecular weight of the polymer impacts nanoparticle size”; thus, a higher polymer molecular weight will result in nanoparticles with smaller size [57]. Accordingly, ES100 molecular weight (150,000 g/mole) [58] was greater than HPMCP HP55 molecular weight (78,000 g/mole) [58], and hence, ES100 nanoparticles (PRNP1) were smaller in size compared to HPMCP HP55 nanoparticles (PRNP2).

The prepared DOX-PRNPs revealed negative ZP, which may give an indication of high stability of the prepared particles because of the low tendency for aggregation. Drug loading was also affected by viscosity of the polymer, where PRNP1, made of ES100 with higher molecular weight, demonstrated lower loading capacity value than that prepared with HPMCP HP55 of lower molecular weight [58]. Upon studying release of DOX-PRNPs, results showed that the type of polymer affects drug release, where PRNP1, formulated using ES100, showed higher release than PRNP2, those formulated using HPMCP HP55; this may be due to that PRNP1 possesses a smaller size compared to PRNP2, resulting in greater surface area. Furthermore, HPMCP HP55 has the ability to dissolve at higher pH than ES100, and both formulations showed more delayed release than the pure drug. Further pharmacodynamic studies were carried out on PRNP2 for the most delayed release.

In the current study, the authors depended on physiochemical characterization based on measuring particle size, ZP, PDI and loading capacity %. In agreement with our study, many recent studies used these values for physicochemical characterization of nanoparticles [59,60,61,62,63].

### 4.2. In Vivo Antitumor and Anti-Angiogenic Activity

Although DOX is a cytotoxic antibiotic with a broad antibacterial range and exerts anti-cancer effects through inhibition of mitochondrial protein synthesis consequently inhibiting cell proliferation, and induces apoptosis of cancer cells [64], its uses in the long term is restricted in that it is attributed to dose-dependent organ toxicities such as cardiotoxicity, nephrotoxicity and hepatotoxicity [65,66,67]. To overcome this obstacle, we suggested a strategy of PRNP development for DOX delivery and compared it to the effect of free DOX. The evolutionary conservation between PRNP design for a loading drug and their potential application for in colon cancer therapy was illustrated in this study.

Nanotherapeutic physicochemical characteristics (for example, size, geometry, surface features, elasticity, stiffness, porosity, composition, targeting ligand, and drug-release kinetics) enhance systemic transport to tumors, increasing permeability and retention and therapeutic results [68].

The current results demonstrated that the proangiogenic factors IL-6, TNF-α and VEGF were elevated in the Di-MH control group compared to saline and were downregulated relatively by the therapeutic remedies. IL-6 is one of the major cancerous media cytokines that controls the pattern of tumor proliferation, apoptosis, metabolism, progression, metastasis, and angiogenesis. Protection of cancer cells by IL-6 from apoptosis and inflammation, DNA damage, antiproliferative, antimetastatic, and antiangiogenic gains of chemotherapy is considered as a major cause of cytotoxic drugs resistance [69,70,71]. IL-6 collectively regulates the tumor proliferation target genes as inflammatory cytokines as TNF-α, and several angiogenic growth factors as VEGF [72]. To mirror the paracrine and autocrine cancer signaling, IL-6 was inoculated with colon cancer cells SW620, wherein Doxycycline conquered the IL-6-induced proliferative and metastatic activities. Moreover, the degree of tumor IL-6 levels is inversely correlated with the cancer prognosis and aggressiveness, through manipulation of extracellular matrix proteins and cancer-associated fibroblasts [73].

VEGF is a potent guide for pro- and neogenesis in cancer environment. It guides the endothelial cell migration and proliferation to the cancer avascular areas. This ensures blood supply to the newly proliferative cancer cells and enhances the generation and stabilization of cancer stem cells [74,75,76]. Previous studies disclosed the angiogenic capabilities of VEGF in Ehrlich solid carcinomas, with correlation to the chemotherapeutic efficacy and prognosis of the tested drug [77,78,79,80]. Herein, DOX-PRNP2 ameliorated the elevated tumor levels of IL-6, TNF-α and VEGF more efficiently than free DOX. These conducted results configure the utility of PRNPs as a useful tool for drug formulation.

Angiogenesis is crucial in tumor invasion; new blood arteries deliver nutrients during tumor cell growth [81] and its inhibition has been considered as a significant target for cancer treatment. CD31 or CD34 are utilized in malignancies as a micro vessel density marker and are taken in to account as a clear indicator of neo-angiogenesis severity [82] found on the surfaces of hematopoietic stem cells, progenitor cells, and endothelial cells of tiny blood vessels [83]. We observed upregulation in the VEGF and CD31 expressions in the colon cancer tissue of DMH mice. Herein, our results are in line with the previous study [84] showing that CD34 expression was augmented in colorectal cancer tissue. Previous evidence has reported that a close relationship between VEGF and malignant tumors with a severe prognosis, such as colon cancer, are linked to high levels of VEGF [85,86,87,88].

However, the observed enhancement of VEGF and CD31 was suppressed by DOX and a higher dose of DOX-PRNP2 showed further inhibition of VEGF that indicated that PRNP2 increased the anti-tumor efficacy of doxycycline on colon cancer. This suggests that encapsulating DOX in PRNP2 enhances its anticancer activity, possibly due to increased transport into tumor cells [89]. These results were consistent with those reported by [90], developing flexible folate-targeted and oxygen/indocyanine green-loaded lipid nanoparticles (FA-OINPs) for dual-mode imaging-guided therapy in ovarian cancer cells, which reduced VEGF and microvessel density expression as well as CD68 expression.

Similarly, doxorubicin- and indocyanine-synthesized green nanoparticles that are loaded on poly(lactic-co-glycolic acid)–lecithin–PEG (DINPs) were evaluated for biologic activity in prior research, which agreed with these findings. In comparison to free medicines, the DINPs had steady spectrum characteristics, greater stability and worthy dispersity. Besides, the DINPs remained in the tumors for a longer period of time [91]. In line with our results, polyester poly(DL-lactide-co-glycolide) (PLGA)/poloxamer nanoparticles loaded with EPAS1 siRNA suppressed pancreatic tumor development and substantially reduced VEGF and CD31 expression [92]. By understanding the mechanisms of PRNPs, several studies were performed utilizing PRNP-based chemotherapy, which revealed that PRNPs loaded with chemotherapies showed increased drug entry to tumors and persistent localization; this leads to significant increases in their anticancer potential [93].

## 5. Conclusions

We are fast gaining a much greater knowledge of the difficulties and potential that cancer nanomedicine presents. This study investigated the significance of the confluence of nanotechnology and tumor biology in overcoming the barrier of chemotherapy. Our study concluded that the DOX-PRNP2 showed better characteristics and drug release % and hence was selected to be tested in the biological study. DOX-PRNP2 inhibited the formation of tumor microvessels and mitigated colon cancer growth in mice to a greater extent compared to the free DOX preparations. Further studies are warranted to confirm greater activity in other animal cancer models. We anticipate that nanomedicines will change the cancer therapy paradigm and that the actual objective of cancer nanomedicine will become a reality in the near future.

## Figures and Tables

**Table 1:** Composition of the prepared DOX-PRNPs with full characterizations.

Formula	Polymer (0.8%)	Particle Size (nm)	PDI	ZP (mV)	Loading Capacity (%)
**PRNP1**	Eudragit S100	245 ± 2.62	0.469 ± 0.05	−16	58.2 ± 1.7
**PRNP2**	HPMC phthalate	478 ± 4.6	0.456 ± 0.1	−19.2	88.2 ± 2.5

DOX-PRNPs: doxycycline polymeric nanoparticles, ZP: Zeta potential, PDI: polydispersity index.

## Data Availability

Data are available from the corresponding author upon request.
